# Laypeople’s Use of and Attitudes Toward Large Language Models and Search Engines for Health Queries: Survey Study

**DOI:** 10.2196/64290

**Published:** 2025-02-13

**Authors:** Tamir Mendel, Nina Singh, Devin M Mann, Batia Wiesenfeld, Oded Nov

**Affiliations:** 1 Department of Technology Management and Innovation Tandon School of Engineering New York University New York, NY United States; 2 Department of Medicine School of Medicine University of California, San Francisco San Francisco, CA United States; 3 Department of Population Health Grossman School of Medicine New York University New York, NY United States; 4 Department of Management and Organizations Stern School of Business New York University New York, NY United States

**Keywords:** large language model, artificial intelligence, LLMs, search engine, Google, internet, online health information, United States, survey, mobile phone

## Abstract

**Background:**

Laypeople have easy access to health information through large language models (LLMs), such as ChatGPT, and search engines, such as Google. Search engines transformed health information access, and LLMs offer a new avenue for answering laypeople’s questions.

**Objective:**

We aimed to compare the frequency of use and attitudes toward LLMs and search engines as well as their comparative relevance, usefulness, ease of use, and trustworthiness in responding to health queries.

**Methods:**

We conducted a screening survey to compare the demographics of LLM users and nonusers seeking health information, analyzing results with logistic regression. LLM users from the screening survey were invited to a follow-up survey to report the types of health information they sought. We compared the frequency of use of LLMs and search engines using ANOVA and Tukey post hoc tests. Lastly, paired-sample Wilcoxon tests compared LLMs and search engines on perceived usefulness, ease of use, trustworthiness, feelings, bias, and anthropomorphism.

**Results:**

In total, 2002 US participants recruited on Prolific participated in the screening survey about the use of LLMs and search engines. Of them, 52% (n=1045) of the participants were female, with a mean age of 39 (SD 13) years. Participants were 9.7% (n=194) Asian, 12.1% (n=242) Black, 73.3% (n=1467) White, 1.1% (n=22) Hispanic, and 3.8% (n=77) were of other races and ethnicities. Further, 1913 (95.6%) used search engines to look up health queries versus 642 (32.6%) for LLMs. Men had higher odds (odds ratio [OR] 1.63, 95% CI 1.34-1.99; *P*<.001) of using LLMs for health questions than women. Black (OR 1.90, 95% CI 1.42-2.54; *P*<.001) and Asian (OR 1.66, 95% CI 1.19-2.30; *P*<.01) individuals had higher odds than White individuals. Those with excellent perceived health (OR 1.46, 95% CI 1.1-1.93; *P*=.01) were more likely to use LLMs than those with good health. Higher technical proficiency increased the likelihood of LLM use (OR 1.26, 95% CI 1.14-1.39; *P*<.001). In a follow-up survey of 281 LLM users for health, most participants used search engines first (n=174, 62%) to answer health questions, but the second most common first source consulted was LLMs (n=39, 14%). LLMs were perceived as less useful (*P*<.01) and less relevant (*P*=.07), but elicited fewer negative feelings (*P*<.001), appeared more human (LLM: n=160, vs search: n=32), and were seen as less biased (*P*<.001). Trust (*P*=.56) and ease of use (*P*=.27) showed no differences.

**Conclusions:**

Search engines are the primary source of health information; yet, positive perceptions of LLMs suggest growing use. Future work could explore whether LLM trust and usefulness are enhanced by supplementing answers with external references and limiting persuasive language to curb overreliance. Collaboration with health organizations can help improve the quality of LLMs’ health output.

## Introduction

Search engines, such as Google, democratized access to health information and changed the dynamics of the patient-provider relationship, with 72% of internet users in the United States looking on the web for health information [1-4]. Clinicians and health organizations initially had mixed reactions, including some early efforts to discourage patients from searching for health information on the web because of concerns that web-based information could make them misinformed or anxious [5-7]. Patients’ use of internet-based health information enhances their understanding and their ability to manage their health conditions [8,9]. Over time, clinicians and health organizations started to partner with search engine companies to improve content reliability. For example, the World Health Organization has worked with Google to ensure that COVID-19 searches yield evidence-based information [10].

Large language models (LLMs) have the potential to replace internet searches for clinicians and patients. LLMs, such as ChatGPT, have demonstrated promising performance in clinical decision-making [11] and diagnosis [12]. Numerous studies have explored doctors’ evaluation of LLMs as an information resource and diagnostic aid [13-18] and some research has begun to consider laypeople’s attitudes toward and use of these tools, such as concerning ethical considerations, whether they are differentiable from doctor’s responses, and the accuracy of symptom-checkers [19-21]. LLMs can potentially make information accessible to patients in more specific and personalized ways, but previous studies have yet to consider patient use of LLMs in comparison to search engines for health-related questions.

We surveyed laypeople in the United States to compare the use of LLMs and search engines for health queries, the types of health queries posed, and attitudes toward interactions with these tools.

## Methods

### Recruitment Procedures

US participants aged older than 18 years were recruited from Prolific, a web-based research participant platform, in February 2024. We programmed the survey questions in Qualtrics. The invitation in Prolific included the purpose and a brief description of this study, a link to the Qualtrics survey, and it informed participants that survey completion would take approximately 5 minutes for study 1 and 5-10 minutes for study 2. Participants completed study 1 in 1.21 (SD 1.04) minutes on average. Participants completed study 2 in 10.01 (SD 7.02) minutes on average. All questionnaires are presented in Multimedia Appendix 1.

### Ethical Considerations

This study was reviewed and approved by New York University’s institutional review board (IRB-FY2024-8278). Participants were presented with an informed consent form including a description of their role as participants in a research study to learn more about patterns of using search engines and LLMs for health queries. If they agreed to participate in this study, they were asked to complete a questionnaire about their experience using a search engine and an LLM for health questions. The research involves no more than minimal risk of harm to participants. At the end of the survey, participants were asked to provide a limited amount of personal data (Prolific ID) to provide compensation and asked about their willingness to participate in the second study if they qualified. Data were stored in password-protected computers of the faculty investigators and their assistants. Participants received compensation of US $0.25 in study 1 and US $2.50 in study 2.

### Survey Questionnaire

A screening survey (study 1) identified the prevalence of participants’ LLM and search engine use for health queries. Next, study 1 participants who reported using both LLMs and search engines to answer health questions were invited to participate in study 2, in which their use of these tools was compared. Study 2 participants were asked which types of health information they sought, and how the results affected their relationships with their providers. Likert scales from 1 (strongly disagree) to 7 (strongly agree) were used to evaluate the effect of LLMs and search engines on participants’ relationships with health care providers.

Participants were asked about LLMs’ versus search engines’ perceived usefulness and ease of use. These two measures represent people’s perception of whether a system would help them perform their tasks and whether the system is easy or difficult to use [22]. Trustworthiness is the extent to which people trust and rely on LLMs or search engines to answer health questions [23]. Relevance is the extent to which participants consider the source as offering output relevant to their health needs. We also asked whether the source’s results were perceived to be biased or benefit advertisers. Finally, we asked about participants’ reactions to LLM and search engine query results [24] and their perceptions of anthropomorphism [25]. Likert scales from 1 (strongly disagree) to 7 (strongly agree) were used to evaluate attitudes toward LLMs and search engines. A separate Likert scale ranging from 1 (not at all) to 7 (extremely) was used for items assessing participants’ feelings about using LLMs and search engines. Finally, we asked about demographics: age, gender, education, income, use of LLMs for health, health status, and perceived tech skills. Perceived technical skills assessed participants’ perceptions of their smartphone skills (1: not at all skilled to 7: expert; Multimedia Appendix 1).

### Statistical Analysis

We compared the demographics of LLM users and nonusers using logistic regression. We used ANOVAs followed by a Tukey post hoc test to compare the frequency of use of LLMs and search engines for each of the types of health information they sought. Using the Shapiro-Wilk test, we observed a nonnormal distribution for perceived usefulness, ease of use, relevance, bias, trustworthiness, feelings, perceptions of benefit to advertisers, and perceptions of anthropomorphism. Therefore, paired samples of Wilcoxon tests were used to compare the effects of LLMs and search engines on these perceptions.

## Results

### Overview

In total, 2002 participants responded to study 1 (see demographic information in Table 1). Furthermore, 300 randomly selected study 1 participants who reported using both LLMs and search engines for health queries completed study 2. Further, 281 participants were retained after indicating the use of LLMs for health-related queries and passing attention checks (see Table 1 for demographic information).

**Table 1:** Demographic characteristics of respondents in study 1 and study 2, with study 2 participants being a subset of those from study 1.

Variable		Study 1	Study 2
Respondents, n		2002	281
Age (years), mean (SD)		39.4 (13)	40.43 (13.17)
**Gender, n (%)**			
	Male	904 (45.2)	161 (57.3)
	Female	1045 (52.2)	112 (39.9)
	Other	53 (1.6)	8 (2.8)
**Race, n (%)**			
	Asian	194 (9.7)	35 (12.5)
	Black	242 (12.1)	46 (16.4)
	Hispanic	22 (1.1)	3 (1.1)
	White	1467 (73.3)	187 (66.5)
	Other	77 (3.8)	10 (3.6)
**Education**			
	High school or less	578 (28.9)	72 (25.6)
	Associate degree	249 (12.4)	39 (13.9)
	Bachelor’s degree	847 (42.3)	125 (44.5)
	Graduate degree	328 (16.4)	45 (16)
**Income (US $)**			
	Less than 50,000	715 (35.7)	106 (37.7)
	50,000-99,999	694 (34.7)	103 (36.7)
	More than 100,000	518 (25.9)	72 (25.6)
	Unknown	75 (3.7)	0 (0)
**Insurance, n (%)**			
	Private insurance	1166 (58.2)	159 (56.6)
	Public insurance	592 (29.6)	92 (32.7)
	No insurance	192 (9.6)	25 (8.9)
	Other insurance	52 (2.6)	5 (1.8)
**Using LLMs^a^ for health, n (%)**			
	Yes	642 (32.1)	281 (100)
	No	1360 (67.9)	0 (0)
**Using search engines for health, n (%)**			
	Yes	1913 (95.6)	281 (100)
	No	89 (4.4)	0 (0)
**Health status, n (%)**			
	Excellent	268 (13.4)	44 (15.7)
	Good	1231 (61.5)	158 (56.2)
	Only fair	426 (21.3)	66 (23.5)
	Poor	67 (3.3)	12 (4.3)
	Prefer not to say	10 (0.5)	1 (0.4)
Perceived tech skills, mean (SD)		5.60 (1.05)	5.85 (1.04)

^a^LLM: large language model.

Among the participants from study 2, a total of 218 (77.6%) participants reported using ChatGPT [OpenAI] as their only LLM tool to answer health questions, 48 (17.1%) reported using both ChatGPT and other LLM tools (such as Bard [Google AI] or Copilot [Microsoft]), and only 15 (5.3%) indicated they used other LLM tools without using ChatGPT. For health-related searches, 228 (81.1%) participants used Google, 51 used both Google and other search engines (such as Bing [Microsoft]), and only 2 (<1%) used Bing alone for finding health answers. Therefore, most participants used ChatGPT as their primary LLM tool and Google search to answer health questions.

### Demographic Comparison: LLM Users Versus Nonusers for Health-Related Questions

A logistic regression analysis revealed that men had higher odds (odds ratio [OR] 1.63, 95% CI 1.34-1.99; *P*<.001) of using LLMs for health-related questions compared to women. Individuals who reported their race as Black (OR 1.9, 95% CI 1.42-2.54; *P*<.001) and Asian (OR 1.66, 95% CI 1.19-2.30; *P*<.01) used LLMs for health-related questions more than participants who identified as White. Participants who perceived their health status as excellent (OR 1.46, 95% CI 1.1-1.93; *P*=.01) reported using LLMs for health-related questions more than those reporting good health status. Individuals with higher perceived technical proficiency have a higher likelihood of using LLMs for health-related questions (OR 1.26, 95% CI 1.14-1.39, *P*<.001; see Table S1 in Multimedia Appendix 1).

### LLMs Versus Search Engines

**Figure 1:**
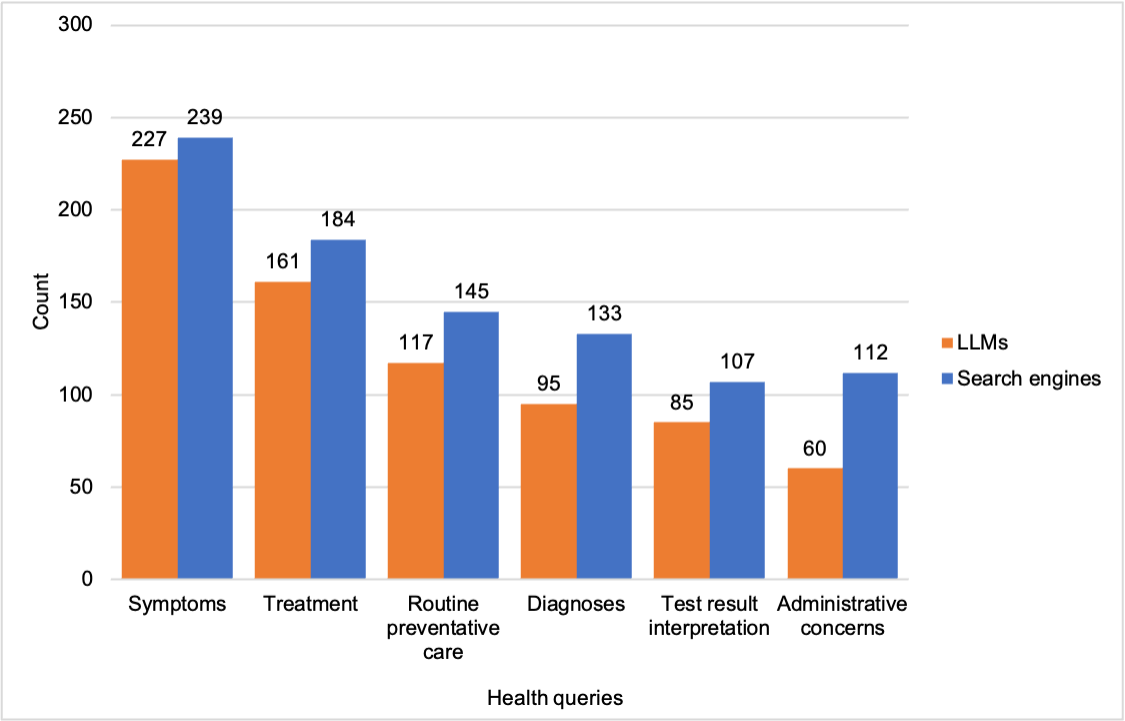
Distribution of health queries between LLMs and search engines among 281 participants who reported using both for health queries. The question asked was “When consulting [Google Search/ChatGPT] for health questions, which questions do you use it to answer?” LLM: large language model.

In the follow-up study (study 2), most participants used search engines first (n=174, 61.9%) to answer health questions, but the second most common first source consulted was LLMs (n=39, 13.9% of participants), followed by doctor’s appointment (n=19, 6.8%), calling their doctor (n=17, 6%), messaging their doctor (n=14, 5%), asking friends (n=7, 2.5%), going to the emergency department (n=6, 2.1%), and other options to answer health questions (n=5, 1.8%). They reported using search engines 6.91 (SD 2.67) times over the past year for health queries, compared to using LLMs 4.7 (SD 2.79) times, and other services fewer times (Figure S1 in Multimedia Appendix 1, *P*<.05). Finally, as shown in Figure 1, there was no difference in the likelihood that participants would use LLMs versus search engines to ask about symptoms (LLM: n=227; search engines: n=239), treatment (LLM: n=161; search engines: n=184), routine preventive care (LLM: n=117; search engines: n=145), diagnoses (LLM: n=95; search engines: n=133), and interpretation of test results (LLM: n=85; search engines: n=107), but they were more likely to use search engines than LLMs for administrative queries (LLM: n=60; search engines: n=112).

**Figure 2:**
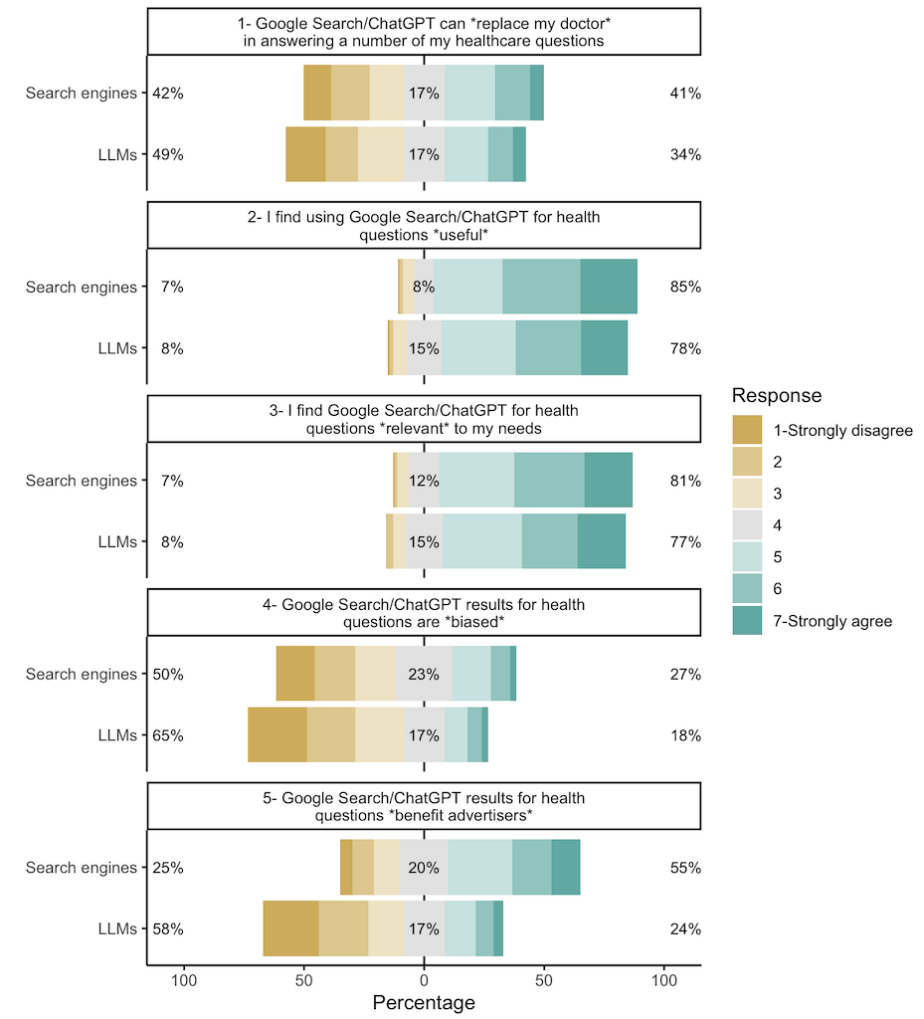
Comparison of LLMs and search engines among 281 participants who reported using both for health queries, using the Wilcoxon signed rank test for replacing "my doctor” (W=9223, *P*=.003), usefulness (W=7214.5, *P*<.01), relevance (W=7117, *P*=.07), result bias (W=9533.5, *P*<.001), and benefits to advertisers (W=20708, *P*<.001). LLM: large language model.

As can be seen in Figure 2, LLMs were perceived to be less useful than search engines for answering health-related questions (W=7214.5, *P*<.01). However, LLMs were also perceived as less biased (W=9533.5, *P*<.001) and less beneficial to advertisers than search engine results (W=20,708, *P*≤.001). Moreover, participants were less likely to view LLMs as being able to replace doctors in answering health care questions than search engines (W=9223, *P*=.003).

**Figure 3:**
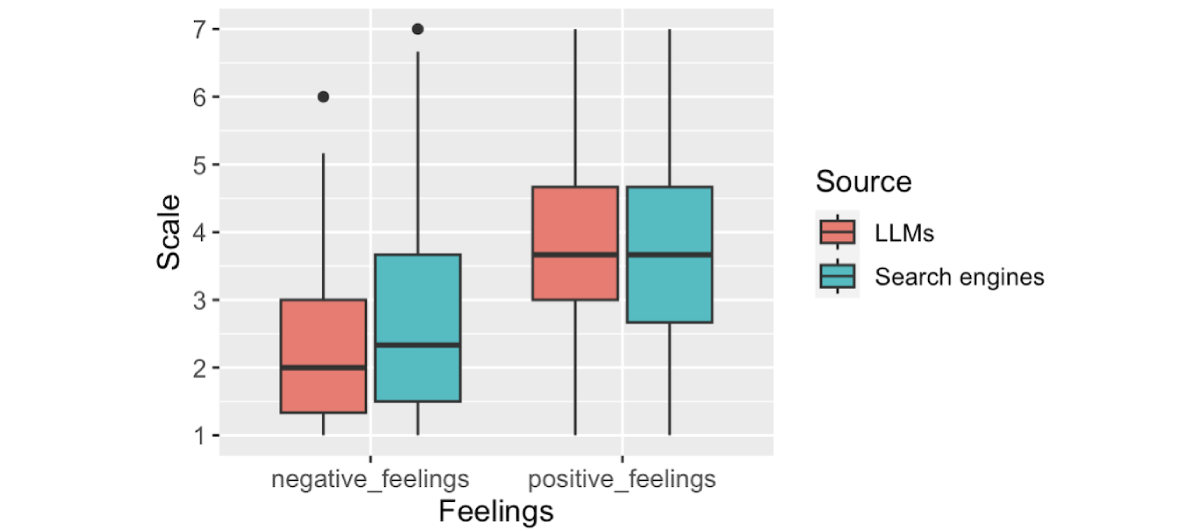
Comparison between LLMs and search engines among 281 participants who reported using both for health queries, based on the Wilcoxon signed rank test for negative feelings (W=19,496, *P*<.001) and positive feelings (W=9773.5, *P*=.01). LLM: large language model.

When searching for health information, results can reassure users but they can also make users more concerned [26]. As shown in Figure 3, LLMs elicited fewer negative feelings in response to the results of their queries than did search engines (W=19,496, *P*<.001). The LLMs also elicited more positive feelings in response to results than search engines did (W=9773.5, *P*=.01), but the median was lower than the midpoint of 4 (W=977.5, *P*<.001). On the other hand, while falling short of statistical significance, search engines were perceived to offer responses more relevant to users’ needs than LLMs (W=7117, *P*=.07). There were no significant differences between ratings of LLMs and search engines in ease of use or trustworthiness of answers (*P*’s>.05). All paired samples of Wilcoxon tests are available in Table S2 in Multimedia Appendix 1. Finally, most participants (n=160, 57%) perceived LLMs as more human than search engines, but a minority perceived search engines as either more human (n=32, 11%) or equivalent to LLMs (n=32, 12%).

## Discussion

### Principal Findings

We compared the use of LLMs, search engines, and other sources for health queries and found that while the most common first source of information participants consulted was search engines, 13.9% of respondents consulted LLMs before any other source. Moreover, they were as likely to use LLMs as search engines for the most common health queries (symptoms, treatment, routine preventive care, diagnoses, and interpretation of test results), though they were more likely to use search engines than LLMs for less common administrative queries. In addition, LLMs elicited fewer negative feelings, such as less distress in response to the information provided, and were perceived as less useful and relevant than search engines. LLMs appeared more human-like, less biased, and less favorable toward advertisers. No significant differences were found between trust in LLMs and trust in search engines.

At this early stage of LLM adoption, participants indicated that LLMs were less useful and relevant than search engines for answering health-related questions. One key reason that search engines provide more useful and relevant health information compared to LLMs could include their greater transparency, such as providing external references to information sources that convey credibility and trustworthiness to users—features that are absent or inaccurate in many popular LLMs. The low perceived usefulness and relevance of LLMs can be attributed to issues such as incorrect statements, hallucinations, and ethical concerns [21,27,28], which can be mitigated through the involvement of domain experts, refining input prompts, and enhancing the fine-tuning of LLMs for specific tasks or domains [29].

Strengths of LLMs relative to search engines that emerged in our study include that LLMs elicited fewer negative feelings, such as less distress in response to the information provided. They also appeared more human-like, less biased, and less favorable toward advertisers. LLMs compared to search engines provide people with a feeling of human-like and personalized attention and understanding, enhancing digital communication that makes information delivery more intuitive. Evidence outside of health care suggests that these features make LLMs as persuasive as humans [30]. LLMs have advanced state-of-the-art performance in generating human-like text based on user health questions [19].

With respect to ease of use, there was no significant difference between LLMs and search engines for health queries. This is notable because search engines have been in regular use for decades while LLMs are a relatively new lay user-facing technology. The fact that respondents perceived that new and unfamiliar technology is as easy to use as an extensively used and highly familiar technology suggests that users may shift their use to LLMs further and relatively quickly.

Prior research has found that trust evaluations shape web-based health information-seeking behavior and compliance with health advice [31-33]. No significant differences were found between trust in LLMs and trust in search engines. A possible explanation for the lack of difference in trustworthiness between search engines and LLMs is the counterbalance between the two dimensions of trust: cognitive trust based on perceived accuracy and competence, and affective trust which depends on believing one’s interests are protected [32]. People perceived that search engines, compared to LLMs, provide more helpful and relevant health information, reinforcing perceived accuracy and competence in health care information. However, search engines were also perceived to prioritize advertisers’ interests more than LLMs did, likely diminishing users’ trust in them. While LLMs excel at engaging with users, their output often lacks interpretation within a medical context [34] and they may sometimes fabricate facts or present incorrect information more convincingly and believably [35]. Future work can further analyze the effects of each trust dimension to better understand perceptions of LLMs.

### Limitations

Our study has some key limitations. All of the participants were recruited from Prolific. Prolific participants tend to be technically proficient and may be more likely to use new technologies such as LLMs than the general population. Additionally, we screened for US residency so our findings may not generalize to other countries and languages. Future studies are needed to evaluate whether these findings generalize to broader populations. Finally, the current inferential analysis aggregates attitudinal reactions across varied types of health queries. A valuable future study would separately inquire about perceptions of technology (eg, usefulness, ease of use, or trust) for different types of health queries to enable more nuanced assessments.

### Conclusion

Overall, most individuals still turn to search engines as their primary source for health-related questions, considering them more useful than LLMs. In the future, people may incorporate LLMs more routinely in their search for answers to health questions. In the case of LLMs, incorporating references and external sources of information would be a beneficial practice to increase trustworthiness. Just as clinicians and health organizations have partnered with search engine companies to enhance the reliability of health-related content [12], similar collaborations could enhance the quality of the health-related information provided by LLMs.

## Appendix

Multimedia Appendix 1 Additional materials.

## Abbreviations

LLM: large language model
OR: odds ratio

## Data Availability

The anonymized data analyzed during this study are available from the corresponding author upon reasonable request.
