# Supplementary material for: Laypeople’s Use of and Attitudes Toward Large Language Models and Search Engines for Health Queries: Survey Study
Source: J Med Internet Res. 2025 Feb 13;27:e64290. doi: 10.2196/64290 (PMC11888097; doi:10.2196/64290)
Supplement: Multimedia Appendix 1 [file jmir_v27i1e64290_app1.docx]

**Questionnaires**

**Survey 1**

1. Have you ever used ChatGPT, Bard, or another Large Language Model (LLM) chatbot? Yes/No.
2. Have you used ChatGPT, Bard, or another Large Language Model (LLM) chatbot for answering health questions?Yes/No.
3. Have you used Google Search or other search engines for answering health questions?
4. Demographics
   1. Age
   2. What is your sex?
   3. What is your race?
   4. What is the highest degree of education you have completed?
   5. What is your household income ranges?
   6. What is your health insurance status?
   7. In terms of your smartphone skills, do you consider yourself to be? (1 - not at all skilled, 7 - expert)
   8. In general, how would you rate your own health?

**Survey 2**

1. Have you ever used ChatGPT, Bard, or another large language model chatbot? Yes/No.
2. Have you used ChatGPT, Bard, or another Large Language Model (LLM) chatbot for answering health questions? Yes/No.
3. Which Large Language Models (LLMs) did you use to answer health questions? (Select all that apply) (ChatGPT/Bard/Other (please specify) )
4. Have you used Google Search or other search engines such as Bing for answering health questions? Yes/No
5. Which search engines did you use to answer health questions? (Select all that apply) (Google Search/Bing/Other (please specify))
6. If you have a question about your health, which of the following sources are you most likely to go to?
   1. Message my doctor via patient care portal
   2. Call my doctor’s office
   3. Schedule a doctor’s appointment
   4. Go to the emergency room or urgent care clinic
   5. Digital Health Retail Services (e.g., Amazon Clinic, Teladoc, GoodRx)
   6. Google or other search engines (e.g., Bing)
   7. ChatGPT or other LLM (e.g., Bard)
   8. Ask friends or family
   9. Other (please specify)
7. In the past year, how many times have you used the following for health questions? (0,1,2,...,10+)
   1. Messaged my doctor via the patient care portal
   2. Called my doctor’s office
   3. Scheduled a doctor’s appointment
   4. Went to the emergency room or urgent care clinic
   5. Digital Health Retail Services (e.g., Amazon Clinic, Teladoc, GoodRx)
   6. Google Search or other search engine (e.g., Bing)
   7. ChatGPT or other LLM (e.g., Bard)

[Note: ChatGPT and Google Search were presented to all participants, and the order was randomized]

From now on, please refer to any search engine (such as Google, Bing, etc.) as a <b>Google Search</b>.

1. When consulting <b>Google Search</b> for health questions, which questions do you use it to answer? (Select all that apply)
   1. Questions about administrative concerns (insurance-related questions, which doctor to go to in my area, etc.)
   2. Questions about routine preventative care (cancer screenings, vaccines, diet and exercise, etc.)
   3. Questions about symptoms (e.g., I have a cough and fever - what is causing them?)
   4. Questions about test result interpretation and understanding (e.g., I got a Hemoglobin A1c value of 8.6% - what does it mean?)
   5. Questions about diagnoses (e.g., I was recently diagnosed with diabetes - what does this mean for me?)
   6. Questions about treatment (e.g., how do I treat a urinary tract infection?)
2. After consulting <b>Google Search</b> for health questions, how often do you change your health habits? (e.g., eating less salt in your diet) (Never, rarely, sometimes, often, always)
3. After consulting <b>Google Search</b> for health questions, how often do you start or change a treatment? (e.g., starting an over-the-counter medication) (Never, rarely, sometimes, often, always)
4. After consulting <b>Google Search</b> for health questions, how often do you contact a healthcare professional (e.g., doctor, nurse, or other healthcare provider) for further advice? (Never, rarely, sometimes, often, always)
5. Indicate your agreement with the following statement: (1-Strongly Disagree, 7-Strongly Agree)
   1. Using Google Search for health advice makes me trust my doctor.
   2. Using Google Search for health advice helps me understand my doctor.
   3. Google Search can replace my doctor in answering a number of my healthcare questions.
6. When seeking answers to health questions, indicate your agreement with the following statement about <b>Google Search</b>: (1-Strongly Disagree, 7-Strongly Agree)
   1. I find using Google Search for health questions useful.
   2. I find Google Search easy to use for health questions.
   3. I find Google Search for health questions relevant to my needs.
   4. I can always rely on Google Search for health questions.
   5. I think that Google Search for health questions performs its role very well.
   6. I think that Google search is competent and effective in answering health questions.
   7. I believe that Google Search is designed to act in my best interest with respect to my health questions.
   8. Select two to indicate that you are paying attention.
   9. I believe that Google Search is designed to understand my needs and preferences with respect to my health questions.
7. After using <b>Google Search</b> to answer health questions, to what extent do you feel: (1-not at all, 7-extremely) (Worried / Confused / Overwhelmed / Reassured/ Depressed / Upset / Distressed / Excited / Supported)
8. Indicate your agreement with the following statement about <b>Google Search</b>: (1-Strongly Disagree, 7-Strongly Agree)
   1. Google search results for health questions are biased.
   2. Google search results for health questions benefit advertisers.

From now on, please refer to any Large Language Model chatbot (such as ChatGPT, Bard, etc.) as a <b>ChatGPT</b>.

Repeating questions 8-15 with chatGPT instead of Google Search.

1. Comparing Google Search and ChatGPT, how much do they have a mind of their own?
   1. Compared to ChatGPT, Google has much more of a mind of its own
   2. The same
   3. Compared to Google, ChatGPT has much more of a mind of its own
2. Other comments about how you use Google (and other search engines) when you have health questions?
3. Other comments about how you use ChatGPT (and other LLMs) when you have health questions?
4. Demographics are the same as in Survey 1.

**Table S1.** Logistic regression analysis for ChatGPT for health questions.

|  | Odd Ratio | 95% CI upper bound | 95% CI lower bound | Sig. |
| --- | --- | --- | --- | --- |
| (Intercept) | 0.06 | 0.03 | 0.13 | P<.001 |
| Sex: Man | 1.63 | 1.34 | 1.99 | *P*<.001 |
| Sex: Non-binary | 0.77 | 0.32 | 1.66 | *P*=.78 |
| Sex: Prefer not to say | 1.18 | 0.30 | 3.99 | *P*=.83 |
| Sex: Not listed | 0.00 | NA | 1.09E+12 | *P*=.97 |
| Race: Black or African American | 1.91 | 1.42 | 2.54 | *P* <.001 |
| Race: Asian | 1.66 | 1.19 | 2.31 | *P* <.01 |
| Race: Hispanic or Latino Americans | 0.89 | 0.31 | 2.24 | *P*=.83 |
| Race: American Indian or Alaska Native | 2.11 | 0.78 | 5.52 | *P*=.14 |
| Race: Native Hawaiian or Pacific Islander | 0.70 | 0.03 | 7.69 | *P*=.97 |
| Race: Other | 1.53 | 0.85 | 2.70 | *P*=.14 |
| Income: $50,000 - $99,999 | 1.05 | 0.82 | 1.35 | *P*=.60 |
| Income: More than $100,000 | 0.86 | 0.65 | 1.15 | *P*=.37 |
| Age | 1.33 | 0.79 | 2.22 | *P*=.30 |
| Education: Associate degree | 1.00 | 1.00 | 1.01 | *P*=.99 |
| Education: Bachelor’s Degree | 1.06 | 0.76 | 1.47 | *P*=.94 |
| Education: Graduate Degree or Above | 1.01 | 0.79 | 1.30 | *P*=.19 |
| Insurance: Public | 1.27 | 0.92 | 1.75 | *P*=.23 |
| Insurance: None | 1.15 | 0.91 | 1.45 | *P*=.25 |
| Insurance: Other | 0.79 | 0.54 | 1.14 | *P*=.61 |
| Health status: Excellent | 1.15 | 0.59 | 2.15 | *P*=.01 |
| Health status: Only fair | 1.46 | 1.10 | 1.94 | *P*=.17 |
| Health status: Poor | 1.17 | 0.91 | 1.50 | *P*=.61 |
| Health status: Prefer not to say | 1.18 | 0.67 | 2.02 | *P*=.97 |
| Skills | 0.81 | 0.16 | 3.18 | *P*=<.001 |

The intercept includes the following categories: woman sex, white race, income < $50,000, high school education or lower, and excellent health status.


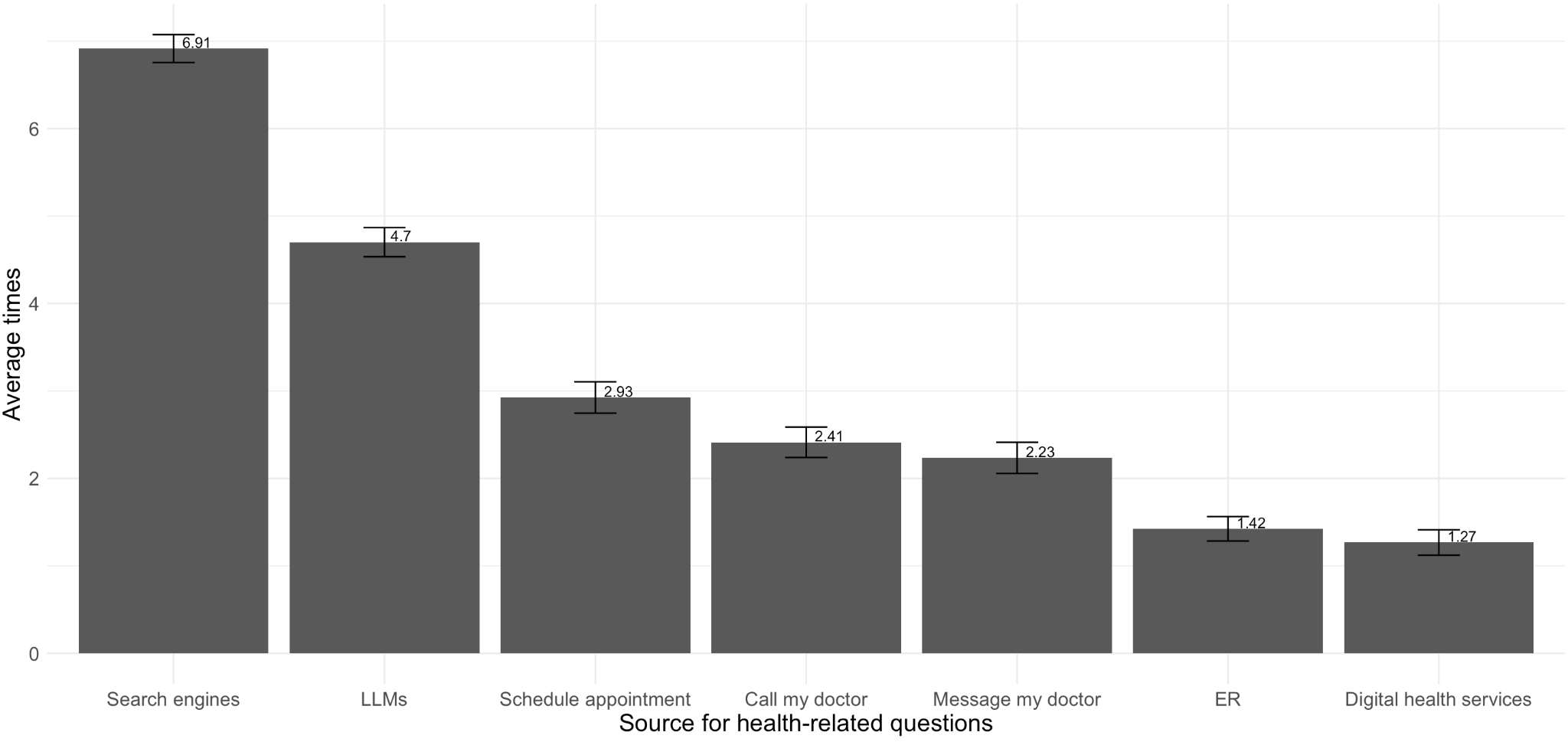


**Figure S1.** Average times referenced last year (and 95% confidence interval in the error bars) for each source.

**Table S2.** Differences between LLMs and search engines using paired samples Wilcoxon tests.

| Variables/Questions | W | *P* |
| --- | --- | --- |
| Using Google Search/ChatGPT for health advice makes me trust my doctor. | 4298 | .42 |
| Using Google Search/ChatGPT for health advice helps me understand my doctor. | 5691.5 | .96 |
| Google Search/ChatGPT can replace my doctor in answering a number of my healthcare questions. | 9223 | .003 |
| Usefulness (I find using Google Search/ChatGPT for health questions useful). | 7214.5 | .001 |
| Ease of use (I find using Google Search/ChatGPTs for health questions easy to use). | 5805.5 | .56 |
| Relevant (I find Google Search/ChatGPT for health questions relevant to my needs). | 7117 | .07 |
| Trustworthiness (5-item; e.g., I can always rely on Google Search/ChatGPT for health questions; Cronbach's alpha is 0.9) | 16558 | .27 |
| Google Search/ChatGPT results for health questions are biased. | 9533.5 | <.001 |
| Google Search/ChatGPT results for health questions benefit advertisers. | 20708 | <.001 |
| Positive feelings (3-item; reassured, excited, supported; Cronbach's alpha is 0.75) | 9773.5 | .01 |
| Negative feelings (6-item; worried, confused, overwhelmed, distressed, depressed, upset; Cronbach's alpha is 0.9) | 19496 | <.001 |

*All items were measured on a 7-point Likert scale.

**To simplify, we referred to the LLM chatbot as ChatGPT and the search engine as a Google search.
